# Feasibility of ^18^F-Fluorocholine PET for Evaluating Skeletal Muscle Atrophy in a Starved Rat Model

**DOI:** 10.3390/diagnostics12051274

**Published:** 2022-05-20

**Authors:** Sun Mi Park, Jisu Kim, Suji Baek, Joo-Yeong Jeon, Sang Ju Lee, Seo Young Kang, Min Young Yoo, Hai-Jeon Yoon, Seung Hae Kwon, Kiwon Lim, Seung Jun Oh, Bom Sahn Kim, Kang Pa Lee, Byung Seok Moon

**Affiliations:** 1 Department of Nuclear Medicine, College of Medicine, Ewha Womans University Seoul Hospital, Ewha Womans University, Seoul 07804, Korea; psm9728@ewhain.net (S.M.P.); eironn02@gmail.com (S.Y.K.); 2 Physical Activity and Performance Institute, Konkuk University, Seoul 05029, Korea; kimpro@konkuk.ac.kr (J.K.); exercise@konkuk.ac.kr (K.L.); 3 Research and Development Center, UMUST R&D Corporation, Seoul 01411, Korea; u-service@naver.com; 4 Seoul Center, Korean Basic Science Institute, Seoul 02841, Korea; jjy0183@kbsi.re.kr (J.-Y.J.); kwonsh@kbsi.re.kr (S.H.K.); 5 Department of Nuclear Medicine, College of Medicine, Asan Medical Center, University of Ulsan, Seoul 05505, Korea; atlas425@amc.seoul.kr (S.J.L.); sjoh@amc.seoul.kr (S.J.O.); 6 Department of Nuclear Medicine, College of Medicine, Ewha Womans University Mokdong Hospital, Ewha Womans University, Seoul 07985, Korea; ckitten@naver.com (M.Y.Y.); haijeon.yoon@gmail.com (H.-J.Y.)

**Keywords:** skeletal muscle atrophy, ^18^F-Fluorocholine, positron emission tomography, MuRF-1, atrogin-1

## Abstract

Imaging techniques for diagnosing muscle atrophy and sarcopenia remain insufficient, although various advanced diagnostic methods have been established. We explored the feasibility of ^18^F-fluorocholine (^18^F-FCH) positron emission tomography/computed tomography (PET/CT) for evaluating skeletal muscle atrophy, as an imaging technique that tracks choline level changes in muscles. Cell uptake in L6 cells by ^18^F-FCH was performed in a complete medium containing serum (untreated group, UN) and a serum-free medium (starved group, ST). Small-animal-dedicated PET/CT imaging with ^18^F-FCH was examined in in-vivo models with rats that were starved for 2 days to cause muscle atrophy. After the hind limbs were dissected, starvation-induced in-vivo models were anatomically confirmed by reverse-transcription polymerase chain reaction to evaluate the expression levels of the atrophy markers muscle RING-finger protein-1 (MuRF-1) and atrogin-1. ^18^F-FCH uptake was lower in the starvation-induced cells than in the untreated group, and in-vivo PET uptake also revealed a similar tendency (the average standardized uptake value (SUV_mean_) = 0.26 ± 0.06 versus 0.37 ± 0.07, respectively). Furthermore, the expression levels of MuRF-1 and atrogin-1 mRNA were significantly increased in the starvation-induced muscle atrophy of rats compared to the untreated group. ^18^F-FCH PET/CT may be a promising tool for diagnosing skeletal muscle atrophy.

## 1. Introduction

The most common body changes that occur with aging are gradual and generalized skeletal muscle disorders that involve an accelerated loss of muscle mass and function [1]. Recently, the US Food and Drug Administration and the medical community defined muscle atrophy as a pathological degenerative disease caused by a reduction in muscle fiber amount, as well as reduced fiber size [2,3]. Diagnosis of musculoskeletal disorders is possible through physical, laboratory, and imaging examinations [4]. Exercise is the only truly reliable therapy to assuage muscle atrophy and, unfortunately, many elderly and/or ill people are unable to participate effectively in physical activity, especially as the muscle deterioration progresses. Thus, early diagnosis of skeletal muscle disorders in the elderly is important and development of analytical techniques for these diagnoses using molecular imaging is urgently required.

Muscle mass is regulated by protein synthesis and degradation [5]. Muscle hypertrophy results from protein synthesis, whereas atrophy is caused by enhanced proteolytic processes, such as calpain and cathepsin activity, as well as ubiquitin proteasome activity, which is marked by skeletal-muscle-specific increased atrogin-1 and muscle RING-finger protein-1 (MuRF-1) mRNA levels [6,7]. Muscle atrophy in the elderly is associated with muscle wasting, sarcopenia, and can occur in cases where starvation/lack of proper diet is an issue. In particular, the physiological response to starvation promotes the breakdown of muscle proteins, thereby resulting in reduced muscle mass. Muscle atrophy is related to the metabolism of carbon nutrients, such as choline. A lack of choline also causes muscle atrophy [8]. Therefore, the detection of fat-related factors or choline may be useful for diagnosing muscle atrophy [9].

Choline is an essential component of cell membrane phospholipids in skeletal muscle cells [10,11]. It binds to the cell membrane in the form of phosphatidylcholine via specific transport mechanisms as choline transporters [12]. Furthermore, choline appears to play a secondary role in inflammatory muscle disease, with anti-fibrotic effects [13]. In terms of functional point, choline is involved in muscle contraction, a precursor to acetylcholine (ACh), a major neurotransmitter in alpha motor neurons [14]. Consequently, it has clinical implications in the finding that healthy people with low serum choline levels show poor physical performance [15]. ^18^F-FCH PET/CT, taken up into cells by choline transporters and subsequently phosphorylated by choline kinase, is mainly used for the detection, staging, and surveillance of malignancies, such as lung and prostate cancers [16,17,18]. However, during muscle atrophy, metabolic function is suppressed, and fat accumulation and protein breakdown are promoted, predicting changes in choline levels. Nevertheless, the use of ^18^F-FCH for the diagnosis of muscle atrophy has not been studied and needs to be verified in various aspects.

Recently, to achieve the diagnosis of sarcopenia, studies on using imaging modalities, dual-energy X-ray absorptiometry (DXA), computed tomography (CT), magnetic resonance (MR), and ultrasound (US), have been reported [19,20,21]. Here, we examined the possibility of diagnosing muscle atrophy using PET, one of the molecular imaging techniques. In the present study, we analyzed the amount of ^18^F-FCH uptake, using both in-vivo and in-vitro models of starvation-induced muscle atrophy, and explored the possibility of its use as a diagnostic biomarker. Based on the results of this analysis, we aimed to provide basic data for diagnosing muscle atrophy via ^18^F-FCH PET in the skeletal muscle.

## 2. Materials and Methods

### 2.1. Cell Culture and Cell Morphology Analysis

L6 cells were cultured in 6-well plates (1 × 10^5^ cells/well), as previously described [22]. The cells were seeded in 1% penicillin–streptomycin and 10% fetal bovine serum in high-glucose Dulbecco’s modified Eagle’s medium at 37 ± 2 °C and 5% CO_2_. After 24 h, the cells were switched to serum-free medium to induce starvation. Cell morphology was observed using light microscopy, and cell size was examined using ImageJ software version 1.52a (US National Institutes of Health, Bethesda, MD, USA). 

### 2.2. Animal Model and Care

Sprague–Dawley rats (male, 8 weeks old) were purchased from Orient Bio (Seongnam, Korea) and maintained in a controlled environment (room temperature, 24 ± 2 °C; humidity, 40 ± 2%; and 12 h light–dark cycle). To allow them to adapt, the rats were housed individually in ventilated cages for 1 week with free access to water and food. Prior to the experiments, the rats were randomly divided into two groups, namely, the untreated group (UN, 283.3 ± 2.68 g, *n* = 6) and the group starved for 48 h (ST, 283.3 ± 2.62 g, *n* = 7). The ST group was provided with free access to water during the period of starvation, whereas the UN group was maintained under standard conditions. The UN group was fasted for 4 h prior to the ^18^F-FCH injection. Before the PET acquisition, the weight of the UN group was 298.0 ± 4.66 g and the ST group was 234.9 ± 5.64 g.

### 2.3. Radiochemistry

^18^F-FCH was prepared on a commercially available automated synthesizer (All-in-One, TRASIS, Ans, Belgium) using a disposable dedicated ^18^F-FCH cassette and a reagent kit. The isolated product with non-decay-corrected radiochemical yield, calculated from trapped radioactivity on a QMA cartridge, was 26.7 ± 1.4% (*n* = 11), with over 99% radiochemical purity as determined by radio-thin liquid chromatography at the end of the synthesis.

### 2.4. Cell Uptake in L6 Cells by ^18^F-Fluorocholine

Cells were cultured by dividing them into two groups, one cultured in complete medium containing serum (untreated group, UN) and the other in serum-free medium (starved group). After 24 h, the medium in each group was replaced with the same medium in which it was incubated with the exception that it contained ^18^F-FCH. Each group of cells (UN and ST) was subdivided into three subgroups. For each treatment (UN and ST), a subgroup was incubated for either 30, 60, or 120 min under the same conditions described until trypsinization. The collected cells were measured for the radioactivity of ^18^F-FCH uptake using a 2480 Wizard^2^ automatic gamma counter (PerkinElmer, Waltham, MA, USA), automatically corrected by the half-life of fluorine-18.

### 2.5. Animal Treatment and Positron Emission Tomography/Computed Tomography Imaging

The rats were anesthetized with 2–3% isoflurane in a 7:3 mixture of N_2_/O_2_ and all rats in each group were administered a single dose of ^18^F-FCH (14.8 ± 2.96 MBq) intravenously, following which PET images were acquired for the diagnosis of muscle atrophy in the untreated and starved rats. After 40 min of conscious radiotracer uptake, rats were anesthetized with 2.5% isoflurane in a 7:3 mixture of N_2_/O_2_, and sequential PET-CT scans were acquired for 20 min using a dedicated small animal PET/CT scanner (NanoPET/CT, Mediso Medical Imaging Systems, Budapest, Hungary). CT scans were used for attenuation correction and anatomical localization of the PET signals. The acquired PET images were reconstructed using the three-dimensional Adjoint Monte Carlo method with scatter and random corrections. The volume of interest (VOI) in both legs (hindlimb) was delineated by the intensely visualized region in the summed image. VOIs were drawn on CT images of individual animals in a slice-by-slice manner. Regional uptake of radioactivity was decay-corrected to the injection time and expressed as the average standardized uptake value (SUV), which was normalized to the amount of radioactivity injected and the animal’s body weight. InterView Fusion software (version 3.03.089.0000, Mediso Medical Imaging Systems, Budapest, Hungary) was used to analyze the data of the SUV in the VOIs after reconstruction and quantification [23].

### 2.6. Histological Analysis

Histochemistry was performed as described previously [22]. Briefly, the gastrocnemius, plantaris, and soleus muscles were obtained by euthanizing the rats (*n* = 3). The muscles were fixed in 4% paraformaldehyde, embedded in paraffin, and serially sectioned into 5 μm slices. The prepared sections were cleared with xylene and hydrated with an ethanol gradient (70, 80, and 90%). To analyze the histological changes, the sections were stained with Masson’s trichrome (MT) solution. The tissues were captured using a microscope (K1-Fluo, Nanoscope systems, Daejeon, Korea) at 200 × magnification. Muscle fiber areas were analyzed using ImageJ version 1.52a (US National Institutes of Health, Bethesda, MD, USA).

### 2.7. Immunohistochemistry

The expression level of choline acetyltransferase (ChAT) was analyzed using immunohistochemistry as previously described [24]. The tissues were sectioned using microtome. The 5 μm thick sections were treated with 3% hydroxy peroxide for 5 min and incubated with 10% normal serum for 1 h at room temperature. The sections were incubated with anti-ChAT (1:200) for 18 h at 4 °C. The following day, the sections were washed and incubated with the corresponding secondary antibody for 1 h at room temperature. The Vectastain ABC Kit was used in accordance with the manufacturer’s instructions. The tissues were incubated with 0.05% 3,3′-diaminobenzidine (DAB), and the slides were stained with hematoxylin. The sections were examined using a light microscope (Olympus BX50; Olympus Co. Ltd., Tokyo, Japan) at 200× magnification.

### 2.8. Reverse-Transcription Polymerase Chain Reaction

The mRNA levels were analyzed as previously reported [25]. The gastrocnemius muscles of untreated and starved rats were harvested, and total RNA was separated from L6 skeletal muscle cells using TRIzol, according to the manufacturer’s instructions. Superscript III First Strand cDNA synthesis kit (Invitrogen, Carlsbad, CA, USA) was used to synthesize cDNA from 1 µg of total RNA. The target gene was amplified using the following primers: MuRF-1 Forward: 5′-GGA GAA GCT GGA CTT CAT CG-3′, MuRF-1 Reverse: 5′-CTT GGC ACT CAA GAG GAA GG-3′; Atrogin-1 Forward: 5′-GAA CAT CAT GCA GAG GCT GA-3′, Atrogin-1 Reverse: 5′-GTA GCC GGT CTT CAC TGA GC-3′; β-actin Forward: 5′-CTA AGG CCA ACC GTG AAA AG-3′; β-actin Reverse: 5′-TCT CCG GAG TCC ATC ACA AT-3′. Amplification involved an initial denaturation at 95 °C for 10 min, followed by 40 cycles of denaturation at 95 °C for 10 s, annealing at 60 °C for 30 s, and extension at 72 °C for 30 s. Relative mRNA levels were calculated using ImageJ, and PCR products were separated by 2.0% agarose gel electrophoresis.

### 2.9. Statistical Analyses

All statistical analyses were performed using Prism software (GraphPad Software version 4.0, La Jolla, CA, USA). Quantitative data are expressed as mean ± standard deviation, and comparisons of quantitative data between the two groups were analyzed using an unpaired t-test. Statistical significance was set at *p* < 0.05.

## 3. Results

To test the hypothesis that tracking changes in choline uptake can confirm the diagnosis of muscle atrophy, we performed in-vitro and in-vivo experiments. For the in-vitro experiments, the cell size changes of L6 cells under a microscope and ^18^F-FCH cell uptake experiment were analyzed in the untreated and starved groups. For in-vivo validation, small-animal-dedicated PET/CT with ^18^F-FCH, and ChAT expression assays were performed.

**Figure 1:**
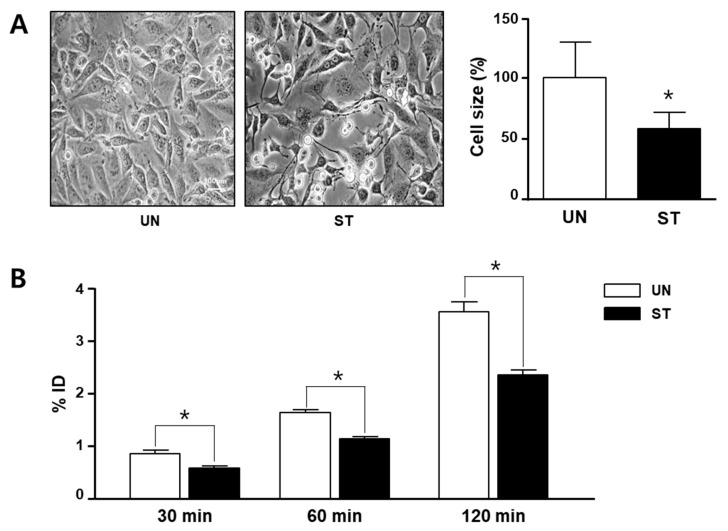
Morphological change and ^18^F-fluorocholine (FCH) uptake in the serum-starved rat skeletal muscle (L6) cell. L6 cells (1 × 10^5^ cells/well) were seeded in 6-well plates and then incubated in the presence or absence of fetal bovine serum supplemented with Dulbecco’s modified Eagle’s medium for 24 h. (**A**) Cell size was analyzed with bright field microscopy (magnification 100×, *n* = 4). The graph indicates the relative cell size from photography. Three random area sizes were analyzed using the ImageJ software. The cell size in the untreated (UN) group is considered 100%. (**B**) L6 cells were incubated with ^18^F-FCH for 30, 60, or 120 min (*n* = 3). The cells were harvested, and uptake of ^18^F-FCH was detected using a gamma counter. The graph is expressed as gamma counter results of the %ID in each condition. All data are expressed as mean ± standard deviation. * *p* < 0.05 vs. the UN group.

Skeletal muscles undergo atrophy during starvation. In fact, L6 cell size decreased and lengthened after 24 h of serum starvation (Figure 1A).

Next, we performed ^18^F-FCH cell uptake to determine whether muscle atrophy could induce choline uptake. As shown in Figure 1B, at 30, 60, and 120 min of incubation, the ST group significantly reduced ^18^F-FCH uptake compared to the UN group (Figure 1B). In addition, ^18^F-FCH cell uptake increased over time, and the difference between the groups also increased significantly.

**Figure 2:**
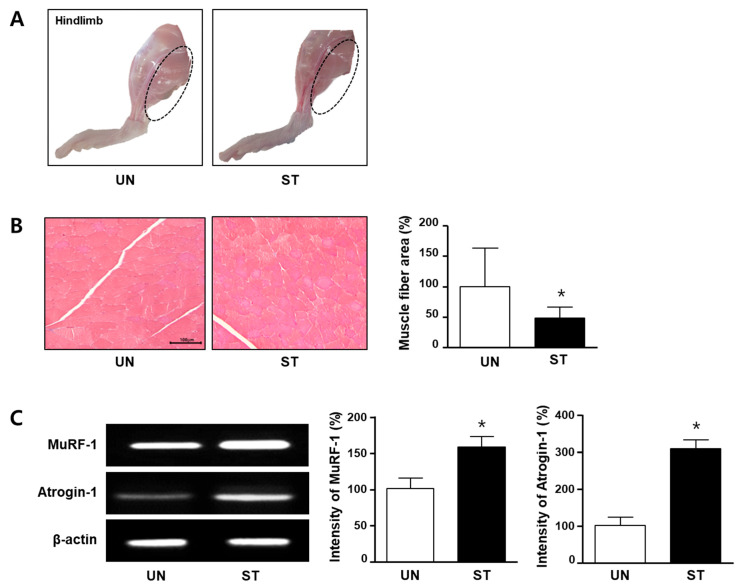
Histological analysis and mRNA expression of muscle RING-finger protein-1 (MuRF-1) and muscle-specific ligases atrophy F-box protein/atrogin-1 in the skeletal muscle of starved rat. The rats were divided into two groups: untreated (UN) and starved-for-48-h (ST) groups. (**A**) The animals were euthanized, and hindlimbs were isolated. The photographs indicate the comparison of muscle mass (dotted circle) in the UN (*n* = 6) and ST groups (*n* = 7). (**B**) The photographs represent muscle fiber area in the UN and ST groups using Masson’s trichrome (MT) stain. The graph expresses the number of muscle fiber area per field of the UN (*n* = 3) and ST groups (*n* = 3). (**C**) The mRNA expression levels of MuRF-1 and atrogin-1 were analyzed using reverse-transcription polymerase chain reaction (*n* = 3). The bands indicate the levels of MuRF-1 and atrogin-1 expressions. Each graph is represented by measuring the bands on the left photograph. The data are expressed as average relative percentages compared with the untreated group ± standard deviations. * *p <* 0.05 vs. the UN group.

Anatomically, the soleus muscle is surrounded by the gastrocnemius. In fasting-induced muscle atrophy in rats, the soleus muscles showed a significantly reduced mass by about 20%, followed by the plantaris and gastrocnemius muscles (Figure 2A, Table S1, *p* < 0.001).

Histological analysis of the same tissue sample under the microscope revealed that the muscle fiber area of the ST group was significantly smaller than that of the UN group. Next, MuRF-1 and atrogin-1, which are indicators of muscle loss, were analyzed at the mRNA level. As shown in Figure 2C, the mRNA expression levels of MuRF-1 and atrogin-1 were significantly higher in the ST group than in the UN group.

**Figure 3:**
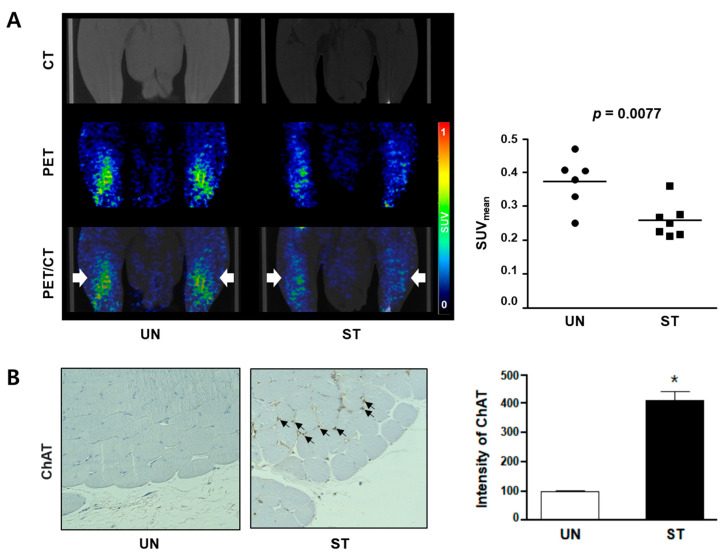
^18^F-Fluorocholine (^18^F-FCH) uptake and choline acetylase expression in skeletal muscle atrophic tissue in rats. (**A**) Choline uptake in the atrophic skeletal muscle was determined using CT and PET with ^18^F-FCH tracer. The volume of interest (VOI; white arrows) indicates both legs (hindlimb). The graph indicates SUV_mean_ in VOI in the UN (*n* = 6) and ST groups (*n* = 7). (**B**) ChAT expression level in gastrocnemius tissue was determined using immunohistochemical staining (*n* = 3). The brown color indicates the positive expression of ChAT (black arrows). The bar graph indicates the relative expression of ChAT. The expression level of ChAT in the UN group is considered 100%. * *p* < 0.05 vs. the UN group. CT, computed tomography; PET, positron emission tomography; ChAT, choline acetyltransferase; SUV_mean_, standardized uptake value mean; UN, untreated; ST, starved.

To assess choline uptake in the skeletal muscle, small-animal PET/CT imaging with ^18^F-FCH was performed in a rat model of muscle atrophy. Thereafter, muscle tissue was extracted, followed by immunohistochemical analysis to evaluate muscle atrophy. As shown in Figure 3A, a clear accumulation of ^18^F-FCH was observed in the muscle tissue of rats, although the degree of analysis of the gastrocnemius and soleus muscles could not be distinguished because of the anatomical proximity.

As evaluated by ^18^F-FCH PET/CT, the quantified mean SUVs of the UN and ST groups were significantly different at 0.37 ± 0.07 and 0.26 ± 0.06, respectively (*p* < 0.001). Figure 3B shows the ChAT expression in gastrocnemius tissue. The relative expression level of ChAT was higher in the ST group than in the UN group (*p* < 0.001).

## 4. Discussion

In the present study, we explored whether ^18^F-FCH PET/CT could be used as a radiotracer for imaging-based diagnosis of muscle atrophy in vitro and in vivo. Through in-vitro analysis, we observed lower ^18^F-FCH uptake in starved L6 cells and reduced uptake in the starvation-induced muscle atrophy of rats. As a result, in the case of muscle atrophy induction, changes in the mRNA expression levels of fat and carbohydrate metabolism were evident, and the gene expression levels of MuRF-1 and atrogin-1 increased. A morphologically altered pattern indicated muscle atrophy. Therefore, these preliminary data suggest this method could be a diagnostic tool for muscle atrophy using ^18^F-FCH PET/CT.

The normal physiological condition of the skeletal muscle and its regulation of muscle protein synthesis/breakdown may be altered due to various pathological conditions, such as cachexia, heart disease, obesity, and aging. Human skeletal muscle reaches its final cell number early in life but decreases in number and size around the age of 30 years [26]. The mechanism of muscle loss proceeds through an imbalance between anabolic and catabolic processes and its effects on intramuscular protein loss. Skeletal muscle protein synthesis can be modulated by exercise with insulin-like growth factor-1, insulin, and testosterone. However, with sarcopenia, the synthesis of skeletal muscle protein is inhibited in various animal models, and in the end, an increase in MuRF-1 and atrogin-1 expression levels leads to protein degradation [7]. Among animal models of muscle atrophy, the fasting-induced model also presents with the expression of MuRF-1 and atrogin-1. Baek et al. reported that reactive oxygen species generation and changes in lipid metabolism were important in a rat model of muscle atrophy induced by fasting for 2 days [22]. Histopathological and molecular marker changes in our results confirmed that muscle atrophy was observed at the cellular level after 48 h of starvation. A hallmark of progressive muscle atrophy is that overexpression of MuRF-1 and atrogin-1 is induced by various factors, leading to a decrease in the size of muscle cells and eventual loss of muscle function [27,28]. The mechanism of atrophy is associated with increased muscle catabolism, which alters carbohydrate-based metabolism, resulting in protein loss and lipid metabolism. Ultimately, the presence or absence of choline indicated changes in the muscle energy metabolism. Our results also showed that the uptake of ^18^F-FCH radiotracer is involved in lipid metabolism and is significant in atrophy modeling, in vitro and in vivo. Therefore, we suggest that ^18^F-FCH PECT/CT is a possible molecular imaging diagnostic tool for muscle atrophy.

Choline plays an important role in maintaining cellular integrity and in the synthesis of neurotransmitters. It plays a role in overall metabolism, as it is involved in cell membrane signaling, fatty acid transmission, brain function improvement, and smooth neurotransmission through methyl group metabolism [9,29,30]. In our recently published study, we confirmed that fasting drastically for 3 days lowered energy metabolism and maintained life by mobilizing fat metabolism rather than carbohydrate metabolism [22]. This appears to have been confirmed by PET/CT image analysis using an ^18^F-FCH radiotracer. There was a higher utilization of fat metabolism compared to carbohydrate use, but this is hypothesized to be due to a rapid decrease in overall energy metabolism. The skeletal muscle has the greatest plasticity in any organ of the human body. An increase in skeletal muscle leads to an increase in basal metabolic rate, and an increase in skeletal muscle decreases energy consumption [31]. According to a study by Jean et al., in 2011, when 8-week-old male rats were induced to fast for 3 days, overall energy consumption and body weight decreased in the rats [32]. This is because fasting causes the loss of both intra-abdominal fat and muscle using energy through the breakdown of fat and protein accumulated in the body.

To answer this question, we performed a PET/CT imaging study using ^18^F-FCH radiotracer. In muscle atrophy, metabolic function is significantly reduced, and the demand for choline increases due to a lack of protein or fat metabolism [33]. Choline deficiency leads to muscle loss, which accelerates fat metabolism and breaks down proteins [34]. In our study, fasting-induced muscle atrophy showed a significant decrease in ^18^F-FCH uptake. In particular, in image acquisition within 60 min, ^18^F-FCH PET/CT showed the tracking of muscle loss. One of the limitations of our study is that it is difficult to distinguish different muscle types, soleus, plantaris, and gastrocnemius, in preclinical studies. Another is that the extent of the quantitative decrease in muscle fiber area (Figure 2B) and the quantitative decrease in SUV values (Figure 3A) are not exactly consistent. ^18^F-FCH PET images, however, indicate that each muscle seems to appear well differentiated in clinical ^18^F-FCH PET images (data not shown). Therefore, we cautiously expect that ^18^F-FCH PET will be clinically useful in the diagnosis of atrophy. Furthermore, since ^18^F-FCH PET is routinely used for the diagnosis of prostate cancer in clinical practice, it has the potential to be easily used in human clinical trials for diagnosing muscular atrophy. This method may also be beneficial to study muscle atrophy conditions for which muscle biopsies are not feasible (i.e., ventilator-induced muscle atrophy).

In addition, we also identified the intensity of ChAT, the enzyme used to synthesize acetylcholine. Diamond et al. assessed the role of muscle mass and function in regulating ChAT activity in mice, postulating that ChAT activity could be an ideal indicator for biochemical measurement of the response to activation or inactivation signals in muscle [35]. In this study, ChAT activity increased with developmental growth of muscle, but not with other forms of muscle growth, i.e., work hypertrophy. Among the methods to arrest muscle growth, the normal development of ChAT activity was not altered by hypophysectomy, and cortisone-induced muscle atrophy did not decrease. On the other hand, in muscle atrophy by tenotomy, ChAT activity was significantly decreased [35]. From this, we also expected unchanged ChAT activity in muscle atrophy derived from starvation; however, it increased instead. Hence, further studies are needed to confirm these results. 

## 5. Conclusions

In conclusion, we propose ^18^F-FCH PET/CT as a potential diagnostic tool for muscle atrophy. In addition, fasting-induced muscle atrophy has been shown to activate MuRF-1 and atrogin-1 and alter the amount of choline in the body. Since our study was focused on molecular imaging, at the cellular and animal levels, its application in other models of muscle atrophy needs to be studied further. 

## Data Availability

The data presented in this study are available from the corresponding author upon reasonable request.

## Supplementary Materials

The following supporting information can be downloaded at: https://www.mdpi.com/article/10.3390/diagnostics12051274/s1, Figure S1: The results of mRNA expression levels of MuRF-1 and atrogin-1 using reverse-transcription polymerase chain reaction (*n* = 3); Table S1: The results of body weight and muscle weight in each group.
