# Supplementary material for: Feasibility of 18F-Fluorocholine PET for Evaluating Skeletal Muscle Atrophy in a Starved Rat Model
Source: Diagnostics (Basel). 2022 May 20;12(5):1274. doi: 10.3390/diagnostics12051274 (PMC9141294; doi:10.3390/diagnostics12051274)
Supplement: Supplementary file 1 [file diagnostics-12-01274-s001.zip › diagnostics-1613982-supplementary.pdf]

Supplementary Materials

Feasibility of ^{18}F-Fluorocholine PET for Evaluating Skeletal Muscle Atrophy in a Starved Rat Model

Sun Mi Park[1,†], Jisu Kim[2,†], Suji Baek[3,†], Joo-Yeong Jeon[4], Sang Ju Lee[5], Seo Young Kang[1], Min Young Yoo[6], Hai-Jeon Yoon[6], Seung Hae Kwon[4], Kiwon Lim[2], Seung Jun Oh[5], Bom Sahn Kim[1,*], Kang Pa Lee[1,3,*], and Byung Seok Moon[1,*]

[1] Department of Nuclear Medicine, College of Medicine, Ewha Womans University Seoul Hospital, Ewha Womans University, Seoul 07804, Korea; psm9728@ewhain.net (S.M.P.); eironn02@gmail.com (S.Y.K.)
[2] Physical Activity and Performance Institute, Konkuk University, Seoul 05029, Korea; kimpro@konkuk.ac.kr (J.K.); exercise@konkuk.ac.kr (K.L.)
[3] Research and Development Center, UMUST R&D Corporation, Seoul 01411, Korea; u-service@naver.com
[4] Seoul Center, Korean Basic Science Institute, Seoul 02841, Korea; jjy0183@kbsi.re.kr (J-Y.J.); kwonsh@kbsi.re.kr (S.H.K.)
[5] Department of Nuclear Medicine, College of Medicine, Asan Medical Center, University of Ulsan, Seoul 05505, Korea; atlas425@amc.seoul.kr (S.J.L.); sjoh@amc.seoul.kr (S.J.O.)
[6] Department of Nuclear Medicine, College of Medicine, Ewha Womans University Mokdong Hospital, Ewha Womans University, Seoul 07985, Korea; ckitten@naver.com (M.Y.Y.); haijeon.yoon@gmail.com (H.-J.Y.)
* Correspondence: bsmoon@ewha.ac.kr (B.S.M.); umustrnd@naver.com (K.P.L.); kbomsahn@ewha.ac.kr (B.S.K.).
†These authors contributed equally to this work.

Figure S1. The results of mRNA expression levels of MuRF-1 and atrogin-1 using reverse-transcription polymerase chain reaction (n = 3).

Table S1. The results of body weight and muscle weight in each group

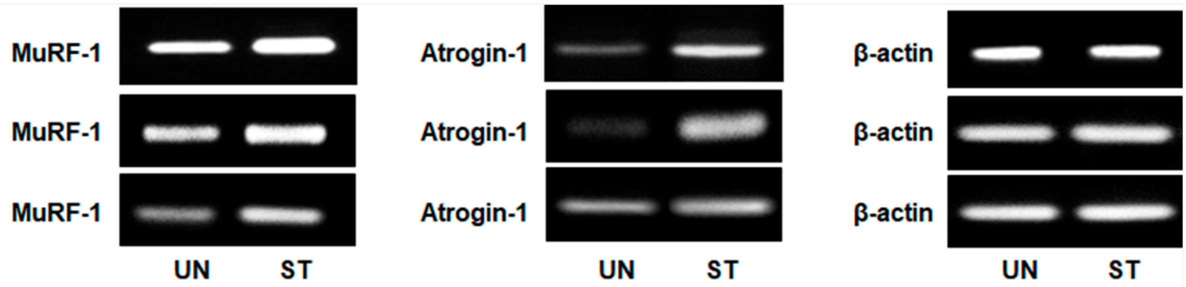

Figure S1. The results of mRNA expression levels of MuRF-1 and atrogin-1 using reverse-transcription polymerase chain reaction (n = 3).

Table S1. The results of body weight and muscle weight in each group

Group	Body weight before experiment (g)	Body weight after treatment (g)	Soleus (mg)	Plantaris (mg)	Gastrocnemius (g)	Total muscle weight (g)
UN	283.3 ± 2.68	298.0 ± 4.66	200.0 ± 5.74	369.5 ± 6.68	1.74 ± 0.60	2.31 ± 0.16
ST	283.3 ± 2.62	234.9 ± 5.64	159.55 ± 7.55	335.8 ± 8.47	1.68 ± 0.95	2.18 ± 0.23

UN: untreated group (n = 6); ST: starvation group (n = 7)
